# Atypical Response Patterns in Renal Cell Carcinoma Treated with Immune Checkpoint Inhibitors—Navigating the Radiologic Potpourri

**DOI:** 10.3390/cancers13071689

**Published:** 2021-04-02

**Authors:** Alvin Wong, Balamurugan Vellayappan, Lenith Cheng, Joseph J. Zhao, Vaishnavi Muthu, Yugarajah Asokumaran, Jia-Li Low, Matilda Lee, Yi-Qing Huang, Nesaretnam Barr Kumarakulasinghe, Natalie Ngoi, Cheng-Nang Leong, Wynne Chua, Yee-Liang Thian

**Affiliations:** 1 Department of Haematology-Oncology, National University Cancer Institute, Singapore 119228, Singapore; vaishnavi_muthu@nuhs.edu.sg (V.M.); yugarajah_asokumaran@nuhs.edu.sg (Y.A.); jia_li_low@nuhs.edu.sg (J.-L.L.); matilda_lee@nuhs.edu.sg (M.L.); yiqing_huang@nuhs.edu.sg (Y.-Q.H.); kumarakulasinghe_nesaretnam@nuhs.edu.sg (N.B.K.); natalie_yl_ngoi@nuhs.edu.sg (N.N.); 2 Department of Radiation Oncology, National University Cancer Institute, Singapore 119228, Singapore; bala_vellayappan@nuhs.edu.sg (B.V.); Cheng_Nang_LEONG@nuhs.edu.sg (C.-N.L.); 3 Department of Diagnostic Imaging, National University Hospital, Singapore 119228, Singapore; lenith_tj_cheng@nuhs.edu.sg (L.C.); wynne_yuru_chua@nuhs.edu.sg (W.C.); yee_liang_thian@nuhs.edu.sg (Y.-L.T.); 4 Yong Loo Lin School of Medicine, National University of Singapore, Singapore 117597, Singapore; jzhaozw@hotmail.com

**Keywords:** renal cell carcinoma, atypical response patterns, pseudoprogression, dissociated response, abscopal response, stereotactic body radiation therapy

## Abstract

**Simple Summary:**

Immunotherapy is now standard treatment for renal cell carcinoma but the interpretation of its efficacy based on routine imaging can be tricky. This is because atypical response patterns are increasingly recognized, giving rise to new scenarios in the patient’s treatment course. We found that many of these atypical patterns can be observed with detailed analyses of the patients’ scans. These atypical patterns challenge the use of conventional and newer tumor response criteria, opening up a range of possibilities for optimizing the efficacy of immunotherapy in renal cell carcinoma.

**Abstract:**

Background: Atypical response patterns have been a topic of increasing relevance since the advent of immune checkpoint inhibitors (ICIs), challenging the traditional RECIST (Response Evaluation Criteria in Solid Tumors) method of tumor response assessment. Newer immune-related response criteria can allow for the evolution of radiologic pseudoprogression, but still fail to capture the full range of atypical response patterns encountered in clinical reporting. Methods: We did a detailed lesion-by-lesion analysis of the serial imaging of 46 renal cell carcinoma (RCC) patients treated with ICIs with the aim of capturing the full range of radiologic behaviour. Results: Atypical response patterns observed included pseudoprogression (*n* = 15; 32.6%), serial pseudoprogression (*n* = 4; 8.7%), dissociated response (*n* = 22; 47.8%), abscopal response (*n* = 9; 19.6%), late response (*n* = 5; 10.9%), and durable response after cessation of immunotherapy (*n* = 2; 4.3%). Twenty-four of 46 patients (52.2%) had at least one atypical response pattern and 18 patients (39.1%) had multiple atypical response patterns. Conclusions: There is a high incidence of atypical response patterns in RCC patients receiving ICIs and the study contributes to the growing literature on the abscopal effect. The recognition of these interesting and overlapping radiologic patterns challenges the oncologist to tweak treatment options such that the clinical benefits of ICIs are potentially maximized.

## 1. Introduction

Immune checkpoint inhibitors (ICIs) have established themselves in the treatment of multiple solid tumors including renal cell carcinoma (RCC) [1,2]. RCC, together with melanoma, is traditionally thought to be a more “immunogenic” tumor, with phenomena such as spontaneous regression well described [3]. Tumor responses to conventional chemotherapy have been assessed using the established RECIST criteria [4]. However, with the onrush of immunotherapy with ICIs, atypical response patterns are now recognized and are a subject of ongoing interest. Several versions of immunotherapy-related imaging response criteria have been developed in an attempt to mitigate the effect of atypical response patterns on tumor response assessment. [5,6]. Pseudoprogression has been the classic atypical response pattern described, but others such as dissociated response are gaining recognition [7]. Practical issues in the clinic include controversies on the indices of immunotherapy efficacy and the decision to treat beyond RECIST-defined progression. We describe the experience in our institution with RCC patients receiving ICIs, looking specifically for atypical response patterns by a detailed analysis of their serial imaging. The growing interest in radio-immunobiology obliged us to include patients receiving concurrent radiotherapy in the search for the elusive abscopal response [8].

## 2. Materials and Methods

### 2.1. Patients

All RCC patients treated with ICIs in our institution with radiologic imaging before and after the start of immune checkpoint inhibitor therapy were included in this retrospective study. We included patients who received single-agent anti-PD1 (nivolumab, pembrolizumab) or combination anti-CTLA4 and anti-PD1 treatment (ipilimumab-nivolumab) [1,2]. We excluded patients who received ICIs in combination with tyrosine kinase inhibitors (TKIs). If the TKI was added onto ICI therapy after an initial ICI-only treatment phase, the imaging was assessed only until the point of addition of TKI. We reviewed all imaging done up to the patient’s death or to the point where TKI was added, irrespective of when the last dose of ICI was given. Patient and tumor characteristics and treatment details were gleaned from the electronic medical records. Ethics approval for the study was obtained under the local Domain Specific Review Board (DSRB 2013/00317).

### 2.2. Atypical Response Patterns Definitions

We reviewed existing definitions of atypical or novel response patterns in the available literature on PUBMED. Taking into account the potential for tumor heterogeneity in response, we looked for these patterns in individual lesions or organ systems in an attempt to capture their full range of radiologic behaviour. Within the ICI-treatment window of assessment for each patient, we reviewed the imaging in relation to key therapy milestones, including radiotherapy (RT) in its various forms and when immune-related adverse events (irAEs) were radiologically demonstrated. Two radiologists (10 and 15 years’ experience in oncologic imaging) reviewed the radiological images on the hospital’s Picture Archiving and Communication System (PACS). One nuclear medicine physician (three years’ experience in oncologic hybrid imaging) also reviewed the positron emission tomography (PET)/computer tomography (CT) images.

We defined pseudoprogression as a decrease or stabilisation of the tumoral elements (including new lesions) that had constituted an initial assessment of progression [9,10]. We looked for pseudoprogression also in individual lesions and whether “serial” pseudoprogression occurred in the same or different lesions at different times [11]. Dissociated response was defined as the concomitant decrease in certain tumoral elements and increase in other elements [9]. We included here the category of “mixed response with new lesions”, which has been described in RCC [12], as well as mixed stable and progressing or regressing lesions [13]. For patients on ICI who subsequently went on to get local therapy for an enlarging or symptomatic lesion, e.g., stereotactic body radiation therapy (SBRT), we looked for the abscopal effect on subsequent imaging—defined as regression of previously stable or enlarging tumors that were outside of the irradiated field. We defined late response as shrinkage or reduction in tumor occurring after an initial period of stability (or progression) of at least 3 months, inferring from the similar categories described by Wolchok et al. for melanoma, and de Velasco et al. for RCC [10,12]. We also looked for any cases of durable response after cessation of ICI [5,12]. We arbitrarily defined this as clinical and radiologic disease control for more than 6 months after cessation of ICI. We did not consider hyperprogression as a separate phenomenon, given the controversies surrounding it [5]. We did not differentiate between target and non-target lesions, and non-measurable tumors were also taken into account in order to elucidate the full variety of serial radiologic appearances.

### 2.3. Diagnostic Imaging

Computer tomography (CT) scans were acquired on 64- or 128-multidetector row CT scanners with intravenous administration of iohexol (300 mg iodine/mL, Omnipaque 350) at a dose of 1–2 mLs/kg body weight injected via power injector at a flow rate appropriate to the cannula size. Portal venous phase imaging was performed in a craniocaudal direction with the aid of bolus-tracking, typically after 65–70 s delay (parameters: 120 kVp, 170–350 mAs; collimation, 0.6 mm). If intravenous iodinated contrast was contraindicated, an unenhanced scan or an alternative modality, e.g., magnetic resonance imaging (MRI) or positron emission tomography CT (PET-CT), was performed for follow-up. Routine dataset reconstructions at 5.0 mm section thickness were performed for the CT scan images in axial and coronal planes.

MRI scans were used to evaluate for brain and spinal metastases in symptomatic or high-risk patients. MRIs were performed on a 1.5 T or 3.0 T scanner. Our clinical protocol for an MRI brain included sagittal FSE T1, coronal T2 fluid attenuated inversion recovery (FLAIR), axial FSE T2, axial diffusion-weighted imaging (DWI), axial susceptibility weighted imaging (SWI), post-gadolinium enhanced axial T2 FLAIR and post-gadolinium enhanced T1 axial, coronal, and sagittal sequences. Our clinical protocol for an MRI spine included sagittal fast spin echo (FSE) T1, FSE T2, short tau inversion recovery (STIR), axial FSE T2/GRE sequences, and post-gadolinium enhanced T1 axial and sagittal sequences.

PET/CT scans were performed on an integrated PET/CT system (Biograph mCT, Siemens Healthcare, Munich, Germany). Patients were instructed to fast for at least six hours before intravenous injection of 2 MBq/kg of 18F-fluorodeoxyglucose (FDG). Sixty to eighty minutes later, a low-dose attenuation CT acquisition was performed (120 kV, 50 mA, 5 mm slice thickness) followed by a static 3D PET acquisition with image duration of 60 s per bed position, an axial field of view of 20 cm, and a matrix of 256 × 256. PET images were reconstructed using the ordered subsets expectation maximization (OSEM 3D) iterative algorithm (3 iterations, 21 subsets), with point spread function and time-of-flight correction (ultra-HD PET). Peak-standardized uptake values, normalized by body weight (maximum standardised uptake value, SUVmax), were calculated on the highest uptake site of up to 5 disease sites at baseline and reassessment examinations.

### 2.4. Radiotherapy

Concurrent RT was defined as radiation received within a month of the start of ICI therapy and up to one month after the last dose of ICI-only therapy. Radiotherapy dose and fractionation was decided at the discretion of the treating radiation oncologist. Symptomatic metastases received a course of palliative radiotherapy, using 3D conformal planning, with hypofractionated radiotherapy, ranging from 8Gy in single fraction to 40Gy in 16 fractions. Patients with oligometastatic disease (defined as up to 5 lesions), or oligoprogression, were treated with a regimen consisting of a higher biologically effective dose. This typically consisted of SBRT (for lung, liver, bone, adrenal metastases) or stereotactic radiosurgery (for brain metastases). SBRT was delivered with rigid immobilisation, highly conformal radiotherapy planning, and image-guided treatments over 2 to 5 fractions, with the fraction sizes ranging from 6–18Gy. Oligometastatic lesions near critical structures (such as heart, bronchus, esophagus) were not eligible for SBRT. These were treated with a high dose fractionated approach ranging from 45Gy in 15 fractions to 55Gy in 20 fractions. Patients with brain metastases were also treated with single fraction Gamma Knife Radiosurgery, with doses ranging from 15–25Gy (depending on lesion size).

### 2.5. Satistical Analyses

Patient, tumor, and treatment characteristics were summarized using descriptive statistics. The Kaplan–Meier method was used to determine overall survival. Hazard ratios were computed using Cox-proportional hazards model. The proportional hazards (PH) assumption was verified by examining scaled Schoenfeld residuals and through the quantitative Grambsch–Therneau test. All survival models did not violate the PH assumption.

## 3. Results

### 3.1. Patient, Tumor, and Treatment Characteristics

We screened 50 patients who received ICI-only drug therapy as part of their advanced RCC management (August 2016 to January 2020). Four patients died without serial imaging and were excluded from analysis. Patient, tumor, and treatment characteristics are shown in Table 1. Fifteen patients received combination ipilimumab-nivolumab while 31 patients received single-agent anti-PD1 therapy (nivolumab or pembrolizumab). Majority were of clear cell carcinoma subtype with Fuhrman or International Society of Urologic Pathologists (ISUP) grading 3–4. About a quarter (26.1%) of the patients had sarcomatoid or rhabdoid components. Majority had prior nephrectomy and were treated with ICI-only drug systemic therapy in the 1st and 2nd-line settings. Most patients (80.4%) were classified as intermediate or poor risk by the International Metastatic RCC Database Consortium [14]. More than half the patients (56.5%) received concurrent radiotherapy, which was mostly delivered as SBRT or GKS. Median survival from initiation of ICI was 29 months (95% CI 25-NR months). The median interval from commencement of ICI treatment to the first assessment scan was 9 weeks (range 2 to 21 weeks). CT scans were done in most patients. PET-CT scans were also done in four patients.

### 3.2. Atypical Response Patterns

**Figure 1:**
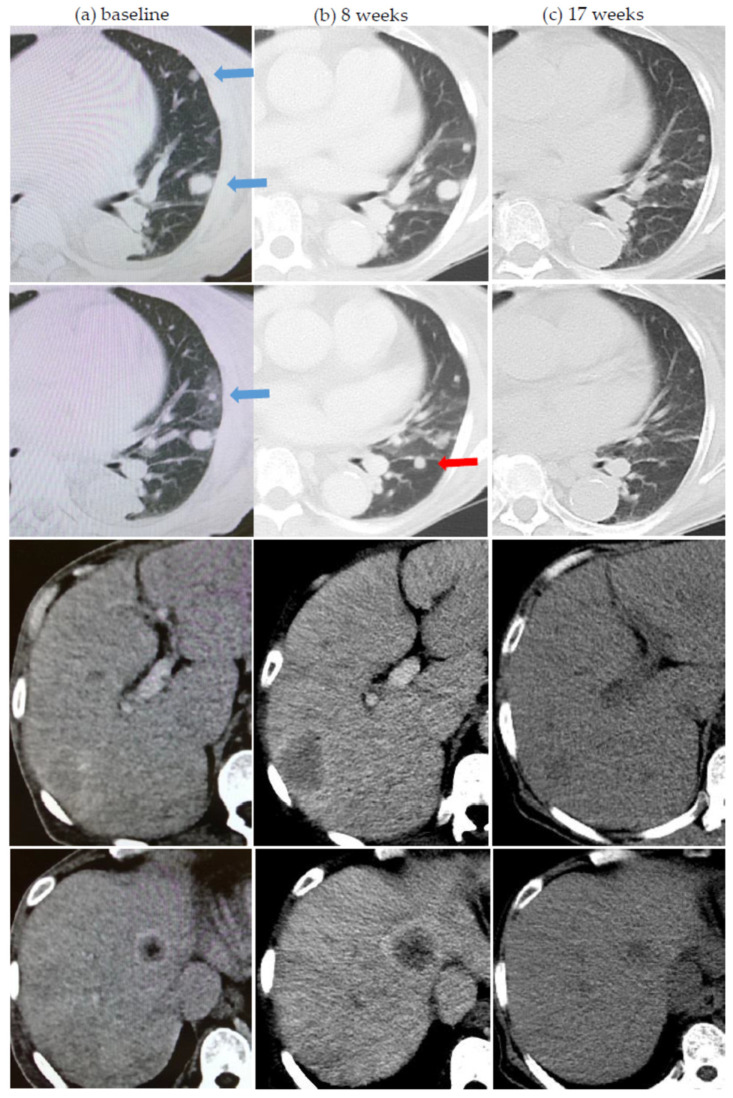
Two atypical response patterns in a 73 year-old lady on first-line nivolumab for metastatic renal cell carcinoma. (**a**) Baseline computer tomography (CT) scan showing lung and liver metastases before starting single-agent nivolumab. (**b**) First assessment CT scan after 8 weeks showing dissociated response—improvement and stability in lung metastases (blue arrows in baseline scan) but obvious worsening of liver metastases. (**c**) Second assessment CT scan at 17 weeks showing continued improvement of lung metastases and now also improvement of liver metastases indicating prior pseudoprogression. On further scrutiny, one small lung lesion (red arrow) had also undergone pseudoprogression.

About half the patients (24, 52.2%) had at least one atypical response pattern observed, while more than a third of patients (18, 39.1%) had multiple atypical response patterns observed. Pseudoprogression, dissociated responses, and abscopal responses were seen in 15 (32.6%), 22 (47.8%), and 9 (19.6%) patients, respectively. Late responses were found in five (10.9%) patients and two (4.3%) patients had durable control of disease after cessation of ICI therapy. Results are summarized in Table 2. Figure 1 shows a patient with dissociated responses (inter- and intra-organ) shown in the first assessment scan and pseudoprogression demonstrated after the second assessment scan.

**Figure 2:**
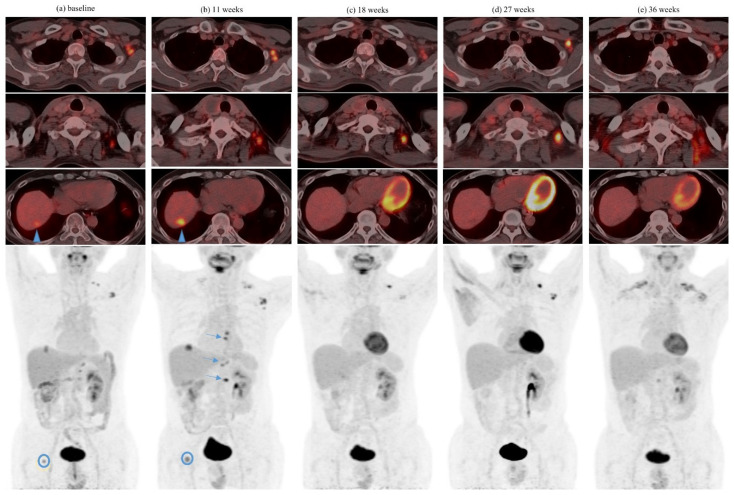
Serial pseudoprogression (inter- and intra-tumoral) in a 54 year-old gentleman on ipilimumab-nivolumab for metastatic renal cell carcinoma. (**a**) Baseline FDG positron emission tomography (PET)/CT demonstrates FDG-avid left supraclavicular and axillary lymphadenopathy, an FDG-avid liver dome metastasis (arrowhead), as well as a focal FDG-avid metastatic deposit in the right femoral head (blue circle). (**b**) Assessment at 11 weeks showing apparent disease progression with an increase in FDG-avidity of left axillary adenopathy, hepatic metastasis, and right femoral head metastasis as well as new FDG-avid mediastinal and upper abdominal adenopathy (arrows on maximal intensity projection (MIP) image). (**c**) Assessment at 18 weeks demonstrates metabolic resolution of mediastinal and upper abdominal lymphadenopathy, hepatic, and right femoral head metastasis. There is an interval decrease in FDG-avidity of left axillary adenopathy but an increase in left supraclavicular adenopathy. (**d**) Reassessment at 27 weeks showing further increase in FDG-avidity of left supraclavicular adenopathy and a recurrent increase in FDG-avidity in the axillary adenopathy. (**e**) Reassessment at 36 weeks showing decrease in FDG-avidity of left axillary lymphadenopathy and metabolic resolution of left supraclavicular lymphadenopathy. Physiological brown fat FDG uptake is seen in the supraclavicular regions.

Among patients with pseudoprogression, serial pseudoprogression was observed in four (8.7%) patients. Serial pseudoprogression was seen comparing metastases of different organs, as well as among the different metastases of the same organ. One patient had both serial pseudoprogression in different lymph nodes as well as in the same lymph node (Figure 2). Symptomatic pseudoprogression occurred in one (2.2%) patient.

### 3.3. Abscopal Response Patterns

**Figure 3:**
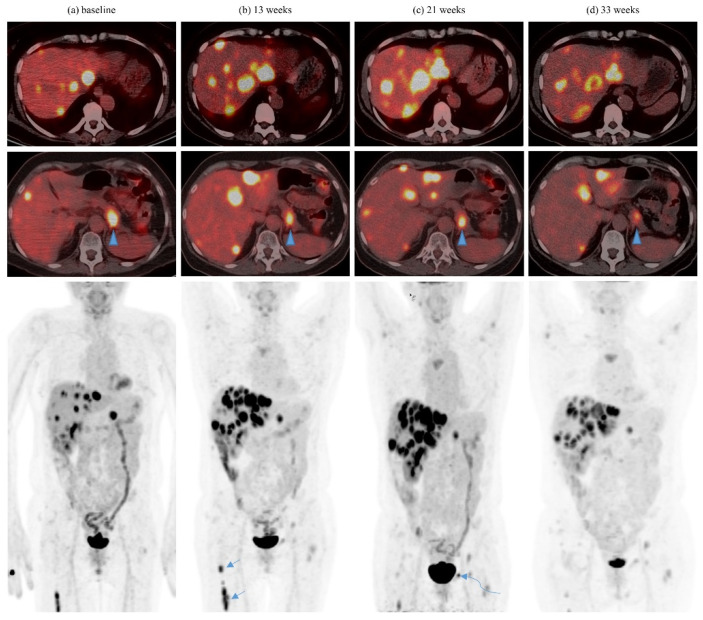
Abscopal response in a 55 year-old lady on pembrolizumab for metastatic renal cell carcinoma. (**a**) Baseline FDG PET/CT demonstrates multiple FDG-avid liver metastases, an FDG-avid deposit in the distal pancreas (arrowhead), and FDG-avid bone metastasis involving the right femur (arrow). (**b**) Assessment at 13 weeks showing dissociated response with an increase in the number, size, and FDG-avidity of liver metastases as well as an increase in the extent and FDG-avidity of the right femoral shaft metastasis but a decrease in size and FDG-avidity of the distal pancreatic metastasis. (**c**) Assessment at 21 weeks demonstrates the abscopal effect after single fraction radiation (8Gy) to the right femur (at 13 weeks) with a decrease in FDG-avidity of a few liver metastases. The distal pancreatic lesion shows relative stability in FDG-avidity. (**d**) Assessment at 32 weeks after another course of radiation (SBRT, 27Gy in three fractions) to the left pubis (at 25 weeks) reveals a more dramatic abscopal effect. There is a remarkable decrease in the size and FDG-avidity of the liver metastases and the distal pancreatic metastasis.

**Figure 4:**
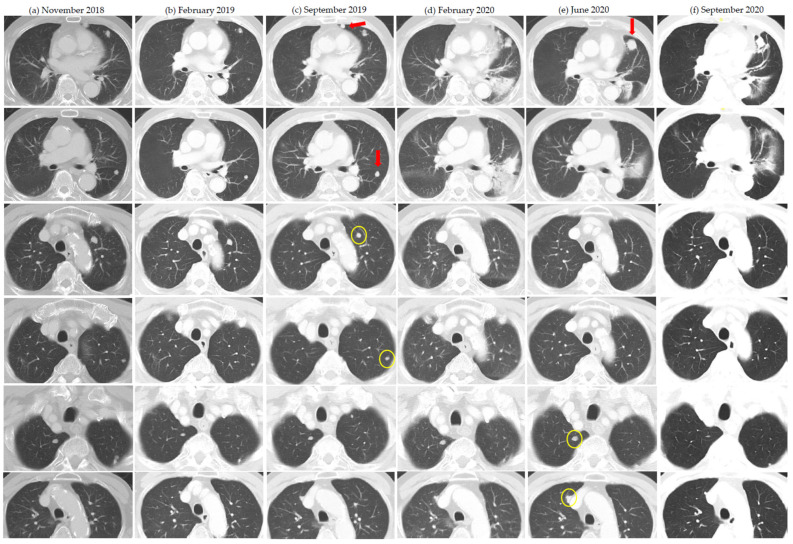
Serial abscopal responses in an 84 year-old man receiving second-line nivolumab for metastatic renal cell carcinoma since May 2018. (**a**–**f**) Serial CT scans of lung metastases from November 2018 to September 2020. Red arrows indicate lesions that received radiation. Panel (**c**) lesions received stereotactic body radiation therapy at 42Gy in five fractions (anterior lesion) and 54Gy in three fractions (posterior lesion), both in October 2019. Panel (**e**) lesion received 25Gy in five fractions in July 2020. Yellow circles indicate out-of-field lesions that showed abscopal pattern of response.

Of the nine patients in whom an abscopal pattern of responses were seen, seven had concurrent radiation and two had their radiation at least 1 month after the last dose of ICI. Most patients received at least 6Gy per fraction with SBRT. The abscopal pattern was seen twice in each of two patients who received multiple courses RT/SBRT. One patient with serial abscopal responses also had prior post-nephrectomy mixed response with spontaneous regression of lung metastases. Another patient had abscopal response after developing severe cytokine release syndrome after two of five planned fractions (10Gy in two fractions delivered) were given about 2 months after the last dose of ICI. In most patients, isolated abscopal responses were seen in one to two lesions, while two patients had more global abscopal responses in specific organs (lung and liver). For the lesions concerned, most had at least two prior scans with stability or progression demonstrated. Table 3 summarizes the patients with observed abscopal pattern of responses. See Figure 3 and Figure 4. 

### 3.4. Other Interesting Immune Phenomena

We observed other interesting and associated immune phenomena. One patient had a prior abscopal effect to radiation while on first-line pazopanib. She achieved durable disease control (more than 1 year and ongoing) after cessation of single-agent nivolumab—which had attained near complete remission as second-line treatment. Another patient had presented with biopsy-proven bone-only RCC metastases without a renal primary, which had presumably undergone spontaneous regression. He achieved long-term disease control with single-agent nivolumab with the development of recurrent immune-related ileitis. Another patient developed florid immune-related pneumonitis with response in his lung metastases concurrent with the pneumonitis and its subsequent resolution captured on the same scans.

### 3.5. Survival Analyses

Assessment of best tumor responses achieved by standard RECIST 1.1 criteria showed complete response (CR) in three (6.5%), partial response (PR) in nine (19.6%), stable disease (SD) in 13 (28.3%), and progressive disease (PD) in 20 (43.5%) patients, with one patient non-evaluable. The overall survival was significantly shorter in those with RECIST PD versus CR/PR/SD (HR 2.42, CI 1.01–5.81, *p* = 0.042). In exploratory analyses of the 20 patients with RECIST PD, there was a trend to improved overall survival in the subgroups with pseudoprogression, dissociated response, abscopal response, any atypical response pattern, and multiple atypical response patterns. The Supplementary Materials (Figures S1–S6) provide details of these analyses.

## 4. Discussion

We found a high incidence of atypical response patterns in our series of RCC patients. Except for hyperprogression, we encompassed most of the patterns described in the literature to date. We also studied the changes observed in individual lesions without being restricted by RECIST-related definitions. Intentionally, we wanted to document a full range of radiologic behaviour for RCC patients on ICI therapy as would be encountered by radiologists in clinical practice. Borcoman et al. commented that immune-related response criteria developed to better capture benefits of immunotherapy only address the pseudoprogression pattern of response, and do not capture the other patterns of response such as hyperprogression and dissociated response [5]. Tazdait et al. remarked that dissociated responses suggest different responses across organs, but a paucity of data are available as conventional RECIST assessment does not facilitate its discovery. Rather, “deep analysis” of CT images is required [9]. The use of the total tumor burden concept may also preclude the discovery of pseudoprogression in individual lesions. The literature in general remarks on the rarity (<10%) of the incidence of pseudoprogression across tumor types [6,15], but we found the incidence in our RCC patients to be higher (32.6%) and the pooled incidence of the various atypical response patterns even higher. This is because the full range of atypical responses was captured by examining all lesions in detail and including all previously reported definitions of atypical response.

Dissociated response in one patient led to a change in treatment strategy: with complete response in a lung nodule that was deemed not easily amenable to surgery or SBRT, while two other lung nodules did not shrink with ICI. Not only did we add local treatment to this patient as Borcoman suggested [5], but by resecting the two non-responding lung nodules, we rendered the patient free of all evaluable disease for a period of time, taking advantage of the complete response of the previously unresectable lesion. In fact, we routinely considered adding local ablative therapy to solitary or oligoprogressing sites in other selected patients with more indolent biology [16]. Several patients received concurrent SBRT or GKS without changing their ICI treatment regime.

In other patients with more numerous sites of progression yet with other sites that remain controlled on ICI, we considered the option of adding a TKI while maintaining ongoing ICI therapy. Although randomised controlled trials have not been done in this setting, the heterogeneity in biology suggested by dissociated radiologic behaviour lends credence to this approach. Addition of TKI to ICI may also allow for synergistic action [17].

Dissociated responses may eventually lead to serial pseudoprogression. We found four such patients and observed serial pseudoprogression among the metastases of different organs, among the different metastases in the same organ, and also serial pseudoprogression in the same metastatic lesion. We believe this reflects both inter- and intra-tumoral heterogeneity leading to a time-differential in radiologic behaviour [18,19].

We defined the abscopal effect in terms of a sequence of radiologic appearances related chronologically to a localized intervention such as SBRT, accepting the uncertainty that a true abscopal effect in terms of biologic mechanism would not be proven. The intervention of local therapy such as SBRT with subsequent out-of-field responses may be coincident with other atypical response patterns (pseudoprogression, late response), which could also explain the responses in previously stable or progressing lesions [20]. In our series, most of the patients had lesions that were shown to be stable or progressing on at least two prior scans, yet we know that late responses and late pseudoprogressions are possible. We acknowledge that clinical trials are in development and have yet to show definitive benefit with the addition of SBRT [8,21]. However, much remains to be discovered about the optimal timing and dosing of SBRT [20,21,22]. If we did indeed show isolated lesions responding to an abscopal effect of SBRT, perhaps this out-of-field effect could be made more global by irradiating a greater total volume or number of sites of tumors [23,24]. Multi-site SBRT, in combination with pembrolizumab, has been shown to be safe and provide excellent local control [25]. The synergy between ICI and SBRT is demonstrated in this Phase 1 trial, where lesions which were only partially irradiated (due to volume constraints) achieved comparable local control to lesions which were completely ablated. In our series, one patient was rendered without evaluable disease when several of his lung metastases were irradiated, and the only remaining out-of-field metastasis responded subsequently as well. The potential for a true abscopal effect with the addition of high dose radiation will be an active area of ongoing research.

In fact, dissociated responses and pseudoprogression were also seen after SBRT. We realise that there will be an overlap of atypical response patterns, with any single patient having the potential to exhibit multiple patterns, as some of our patients did. The definitions are based on changes in lesion sizes over time, irrespective of the actual underlying mechanism. The varied and complex radiological observations by diligent individual-lesion analysis can add up to a vivid potpourri that make their interpretation thought-provoking.

We did consider hyperprogression as an emerging atypical response pattern but felt that this would require a more in-depth analysis warranting a separate paper. The concept of hyperprogression itself retains controversy, having no consensual definition and with a wide range of frequencies (4% to 29%) described among multiple tumor cohorts [26]. Requirements for capturing this phenomenon include having two scans pre-immunotherapy (ideally during an 8-week washout period from the previous therapy), and an assessment scan after starting immunotherapy [27]. This would exclude patients who are starting immunotherapy in the 1st-line setting and may not have prior sequential scans demonstrating the baseline tumor growth rate, and those who have rapid clinical deterioration after starting immunotherapy with the lack of feasibility to perform another scan [26]. In our series, almost half (47.8%) the patients received ICIs in the 1st-line setting. We had also excluded four patients who deteriorated and died soon after starting ICI therapy without the opportunity to do an assessment scan. The mechanism for hyperprogression could be further contributed by an abrupt discontinuation of prior therapy, with rapid tumor flare a known phenomenon after discontinuation of TKIs [28]. Although Ferrara et al. found that hyperprogression was associated with a worse prognosis than standard progression, yet 10% of hyperprogressors in their study were reclassified as pseudoprogression subsequently [29]. We find that even taking clinical deterioration as part of the criteria for hyperprogression is not without controversy. The patient from our series who would otherwise have been the most obvious candidate for hyperprogression had experienced severe clinical deterioration before pseudoprogression became the apparent pattern [30].

Our series captured cases of “multiple immune phenomena”, with typical or atypical response patterns occurring in patients with prior immune events before the start of ICI treatment and/or subsequent interesting immune-related toxicities. The patient with immune pneumonitis and subsequent resolution concurrent with tumor response in the same organ leaves us wondering if this was more than just a coincidence. One Japanese study found an association between peritumoral pneumonitis and good tumor response to nivolumab in patients with non-small cell lung cancer and melanoma [31].

We included four patients with FDG PET/CT in our series. Analogous to CT criteria, with the increasing recognition of atypical response patterns in PET-based response evaluation in the immunotherapy era, the Positron Emission Tomography Response Criteria in Solid Tumors (PERCIST) [32] has also begun to evolve. Several adapted models have been evaluated in various cancers such as melanoma (imPERCIST5) [33], non-small cell lung carcinoma (iPERCIST) [34], and Hodgkin disease (LYRIC) [35], which attempt to account for atypical response patterns after the initiation of ICI therapy. In our patient with serial pseudoprogression evaluated with FDG PET/CT (Figure 2), the criteria for progressive metabolic disease in the PERCIST guidelines would have been met (appearance of new FDG-avid metastatic lesions, increase in tumor SUV of more than 25%). However, using the criteria adapted from the imPERCIST5, iPERCIST, and LYRIC models, the patient would have been classified as stable metabolic disease, unconfirmed progressive metabolic disease, and indeterminate response, respectively. The latter two would necessitate a reassessment PET/CT which would have confirmed pseudoprogression.

The practical consequences of atypical response patterns include clinical decision making on whether to continue treatment beyond RECIST progression. In RCC, George et al. reported on 36 RCC patients on nivolumab who were treated beyond initial RECIST progression, with 25 (69%) experiencing subsequent tumor reduction or stabilization in target lesion size [36]. In CheckMate 025, 13% of patients who continued nivolumab treatment post-progression experienced ≥ 30% tumor burden reduction from the baseline assessment of first progression [37]. It is by now clear that standard RECIST-defined progression or progression-free survival may not be the best index of ICI treatment efficacy. The era for using immune-related response criteria, e.g., iRECIST in ICI-treatment trials, could be near [9,38,39]. We had previously described our case of symptomatic, “extreme” pseudoprogression with a critical end-of-life situation after starting on ICI with subsequent improvement [30]. Although the common practical advice is to withhold and change therapy if the patient is unwell, prudential judgement should be made in cases where milder symptoms from initial progression may allow for the subsequent demonstration of true pseudoprogression.

The duration of use of ICI could be maximized by local treatment such as SBRT to one or more progressive lesions in a dissociated response, also in an attempt to produce an abscopal effect, especially in oligoprogressors with a more indolent course [16]. Another viable option after dissociated response would be to add a TKI to the ICI, using the combination as the next line of systemic treatment. In those with a clearly indolent biology, there may be the luxury of time to allow for pseudoprogression (or even serial pseudoprogression) and late responses to occur. Durable control after cessation of ICI would allow for reintroduction of ICI at the point of progression or recurrence [40]. Rechallenge with combination anti-CTLA4 and anti-PD1 (e.g., Ipilimumab and Nivolumab) may also be a reasonable option in selected circumstances [41], which we had done with two patients in this series. The oncologist is challenged to maximize the clinical benefit of ICI therapy by his understanding of the atypical patterns of response.

We acknowledge the limitations in this essentially descriptive and retrospective study. Intervals between scans were decided in a real-world setting, taking into account individual practice habits and the expected exigencies of clinical care. This also teaches us that the imaging intervals can affect the likelihood of observing atypical response patterns. A shorter interval to the first assessment scan would facilitate a higher chance of catching pseudoprogression. One patient had no baseline post-nephrectomy scan; hence, any subsequent response could have been a post-nephrectomy effect. Another patient had the prior line of TKI maintained while ICI was added to it on progression, although we felt that the atypical response pattern observed was immunotherapy related. We did not apply strict measurement criteria for the various atypical response patterns. We did not have immunologic biomarker correlates to support the occurrence of true abscopal effects [42].

In RECIST PD patients, we showed a trend towards improved survival for those with atypical response patterns. We acknowledge the exploratory nature of the data, given the small sample size, heterogeneity of variables, and the lack of rigorous definitions used to measure atypical responses. Nonetheless, we feel that our results are consistent with the findings of other authors. Tazdait et al. found that patients with pseudoprogression or dissociated response had higher overall survival than patients with true progression [9]. Of the 20 patients with RECIST PD, four patients (20%) demonstrated subsequent tumor size reductions with treatment beyond progression or pseudoprogression demonstrated. This phenomenon is consistent with findings of larger cohorts [36,37].

## 5. Conclusions

We found a high incidence of atypical response patterns in RCC patients receiving ICI therapy when detailed lesion-by-lesion analysis of serial imaging was done. The recognition of these interesting and overlapping radiologic patterns has implications on response assessment, clinical decision making, and treatment options. The suggestion of an improved prognosis in RECIST PD patients with atypical response patterns should be confirmed in a larger prospective study.

## Figures and Tables

**Table 1:** Patient, tumor, and treatment characteristics.

	N	%
Age (years)	Median 60	Range 42–84
Gender (male)	34	73.9
Race		
Chinese	38	82.6
Malay	4	8.7
Indian	2	4.3
Caucasian	2	4.3
ECOG ^1^ performance status		
0–1	30	65.2
2–3	16	34.8
Histology		
Clear cell	37	80.4
Sarcomatoid/rhabdoid component	12	26.1
Papillary	1	2.2
Fuhrman/ISUP ^2^ 3–4	32	69.6
IMDC ^3^ risk		
Good	9	19.6
Intermediate	26	56.5
Poor	11	23.9
Prior nephrectomy	38	82.6
ICI ^4^ regime		
Ipilimumab-Nivolumab	15	32.6
Nivolumab or Pembrolizumab	31	67.4
Treatment setting		
1st line	22	47.8
2nd line	13	28.2
3rd line and beyond	11	23.9
Concurrent radiation		
Any concurrent radiation	26	56.5
Concurrent SBRT ^5^/GKS ^6^	20	43.5

^1^ ECOG: Eastern Cooperative Oncology Group; ^2^ ISUP: International Society of Urologic Pathologists; ^3^ IMDC: International Metastatic RCC Database Consortium; ^4^ ICI: immune checkpoint inhibitor; ^5^ SBRT: stereotactic body radiation therapy; ^6^ GKS: gamma knife surgery.

**Table 2:** Atypical response patterns observed.

	Ipilimumab-Nivolumab		Nivolumab or Pembrolizumab		Combination or Single-Agent ICI ^1^	
	N	%	N	%	N	%
Patients	15	100	31	100	46	100
Any atypical response patterns	9	60	15	48.4	24	52.2
Pseudoprogression	4	26.7	11	35.5	15	32.6
Serial pseudoprogression	2	13.3	2	6.5	4	8.7
Symptomatic pseudoprogression	0	0	1	3.2	1	2.2
Dissociated response	9	60	13	41.9	22	47.8
Abscopal response	4	26.7	5	16.1	9	19.6
Late response	2	13.3	3	9.7	5	10.9
Durable response after cessation	0	0	2	6.5	2	4.3
Multiple atypical response patterns	6	40	12	38.7	18	39.1

^1^ ICI: immune checkpoint inhibitor.

**Table 3:** Patients with abscopal patterns.

Patient Number	ICI ^1^ Regime	Time of RT ^2^ from Start of ICI (Months)	RT Regime (Dose/Fractions)	Concurrent with ICI	RT Site	Abscopal Response (Site, Number of Lesions)	Number of Prior Scans with Non-response	Other Remarks
1	Ipilimumab + Nivolumab	6	55Gy/20	Yes	Lung	Lung, 1	3	
2	Ipilimumab + Nivolumab	6	25Gy/5	2 months after last dose of ICI	Femur, rib	Nephrectomy bed, 1	3	CRS ^3^ after 2 fractions
3	Ipilimumab + Nivolumab	10 14	36Gy/3 30Gy/5	Yes Yes	Lung Adrenal	Lung, 2 Lung, 1	2 5	1 tumor with abscopal response after initial enlargement post SBRT ^4^
4	Ipilimumab + Nivolumab	12	GKS ^5^ 25Gy	1 month after last dose of ICI	Brain (4 lesions)	Intramuscular, 1	2	
5	Pembrolizumab	3 6	8Gy/1 27Gy/3	Yes Yes	Femur Pubic bone	Liver, several	1	Concurrent axitinib from prior line of treatment maintained
6	Nivolumab	17 26	54Gy/3 42Gy/5 25Gy/5	Yes Yes	Lung Lung	Lung, 2 Lung, 2	5 3	Prior spontaneous regression and dissociated response after cytoreductive nephrectomy
7	Nivolumab	6 7 7	50Gy/20 48Gy/3 48Gy/3	Yes	Lung Lung Lung	Lung, 1	1	
8	Nivolumab	9 12 12	20Gy/5 24Gy/3 GKS 25Gy	Yes	Femur Iliac bone Brain (2 lesions)	Lung, several	2	
9	Nivolumab	5 5	27Gy/3 24Gy/3	Yes	Iliac bone Spine	Lung, several Kidney, 1	2	

^1^ ICI: immune checkpoint inhibitor; ^2^ RT: radiotherapy; ^3^ CRS: Cytokine Release Syndrome; ^4^ SBRT: stereotactic body radiation therapy; ^5^ GKS: gamma knife surgery.

## Data Availability

Data available on request to the corresponding author.

## Supplementary Materials

The following are available online at https://www.mdpi.com/article/10.3390/cancers13071689/s1, Figure S1: Overall survival by RECIST non-progressive disease versus progressive disease (PD), Figure S2: Overall survival in RECIST PD patients by presence or absence of pseudoprogression, Figure S3: Overall survival in RECIST PD patients by presence or absence of dissociated response, Figure S4: Overall survival in RECIST PD patients by presence or absence of abscopal response, Figure S5: Overall survival in RECIST PD patients by presence or absence of any atypical response pattern, Figure S6: Overall survival in RECIST PD patients by presence or absence of multiple atypical response patterns.

## References

[1] Motzer R.J., Tannir N.M., McDermott D.F., Arén Frontera O., Melichar B., Choueiri T.K., Plimack E.R., Barthélémy P., Porta C., George S. (2018). Nivolumab plus Ipilimumab versus Sunitinib in Advanced Renal-Cell Carcinoma. N. Engl. J. Med..

[2] Motzer R.J., Escudier B., McDermott D.F., George S., Hammers H.J., Srinivas S., Tykodi S.S., Sosman J.A., Procopio G., Plimack E.R. (2015). Nivolumab versus Everolimus in Advanced Renal-Cell Carcinoma. N. Engl. J. Med..

[3] Wong A.S., Chong K.T., Heng C.T., Consigliere D.T., Esuvaranathan K., Toh K.L., Chuah B., Lim R., Tan J. (2009). Debulking nephrectomy followed by a “watch and wait” approach in metastatic renal cell carcinoma. Urol. Oncol..

[4] Eisenhauer E.A., Therasse P., Bogaerts J., Schwartz L.H., Sargent D., Ford R., Dancey J., Arbuck S., Gwyther S., Mooney M. (2009). New response evaluation criteria in solid tumours: Revised RECIST guideline (version 1.1). Eur. J. Cancer.

[5] Borcoman E., Kanjanapan Y., Champiat S., Kato S., Servois V., Kurzrock R., Goel S., Bedard P., Le Tourneau C. (2019). Novel patterns of response under immunotherapy. Ann. Oncol..

[6] Borcoman E., Nandikolla A., Long G., Goel S., Le Tourneau C. (2018). Patterns of Response and Progression to Immunotherapy. Am. Soc. Clin. Oncol. Educ. Book..

[7] Humbert O., Chardin D. (2020). Dissociated Response in Metastatic Cancer: An Atypical Pattern Brought Into the Spotlight with Immunotherapy. Front. Oncol..

[8] Xing D., Siva S., Hanna G.G. (2019). The Abscopal Effect of Stereotactic Radiotherapy and Immunotherapy: Fool’s Gold or El Dorado?. Clin. Oncol. R. Coll. Radiol..

[9] Tazdait M., Mezquita L., Lahmar J., Ferrara R., Bidault F., Ammari S., Balleyguier C., Planchard D., Gazzah A., Soria J.C. (2018). Patterns of responses in metastatic NSCLC during PD-1 or PDL-1 inhibitor therapy: Comparison of RECIST 1.1, irRECIST and iRECIST criteria. Eur. J. Cancer.

[10] Wolchok J.D., Hoos A., O’Day S., Weber J.S., Hamid O., Lebbé C., Maio M., Binder M., Bohnsack O., Nichol G. (2009). Guidelines for the Evaluation of Immune Therapy Activity in Solid Tumors: Immune-Related Response Criteria. Clin. Cancer Res..

[11] Ozaki Y., Shindoh J., Miura Y., Nakajima H., Oki R., Uchiyama M., Masuda J., Kinowaki K., Kondoh C., Tanabe Y. (2017). Serial pseudoprogression of metastatic malignant melanoma in a patient treated with nivolumab: A case report. BMC Cancer.

[12] De Velasco G., Krajewski K., Albiges L., Awad M., Bellmunt J., Hodi F., Choueiri T.K. (2016). Radiologic Heterogeneity in Responses to Anti-PD-1/PD-L1 Therapy in Metastatic Renal Cell Carcinoma. Cancer Immunol. Res..

[13] Vaflard P., Paoletti X., Servois V., Pons-Tostivint E., Sablin M.P., Ricci F., Loirat D., Hescot S., Torossian N., Borcoman E. (2019). Dissociated responses in patients with metastatic solid tumors treated with immunotherapy. Ann. Oncol..

[14] Heng D.Y., Xie W., Regan M.M., Warren M.A., Golshayan A.R., Sahi C., Eigl B.J., Ruether J.D., Cheng T., North S. (2009). Prognostic factors for overall survival in patients with metastatic renal cell carcinoma treated with vascular endothelial growth factor-targeted agents: Results from a large, multicenter study. J. Clin. Oncol..

[15] Park H.J., Kim K.W., Pyo J., Suh C.H., Yoon S., Hatabu H., Nishino M. (2020). Incidence of Pseudoprogression during Immune Checkpoint Inhibitor Therapy for Solid Tumors: A Systematic Review and Meta-Analysis. Radiology.

[16] Cheung P., Thibault I., Bjarnason G.A. (2014). The emerging roles of stereotactic ablative radiotherapy for metastatic renal cell carcinoma. Curr. Opin. Support. Palliat. Care.

[17] Rassy E., Flippot R., Albiges L. (2020). Tyrosine kinase inhibitors and immunotherapy combinations in renal cell carcinoma. Ther. Adv. Med. Oncol..

[18] Fisher R., Larkin J., Swanton C. (2012). Inter and Intratumour Heterogeneity: A Barrier to Individualized Medical Therapy in Renal Cell Carcinoma?. Front. Oncol..

[19] Wei X., Choudhury Y., Lim W.K., Anema J., Kahnoski R.J., Lane B., Ludlow J., Takahashi M., Kanayama H., Belldegrun A. (2017). Recognizing the Continuous Nature of Expression Heterogeneity and Clinical Outcomes in Clear Cell Renal Cell Carcinoma. Sci. Rep..

[20] Trommer M., Yeo S.Y., Persigehl T., Bunck A., Grüll H., Schlaak M., Theurich S., von Bergwelt-Baildon M., Morgenthaler J., Herter J.M. (2019). Abscopal Effects in Radio-Immunotherapy-Response Analysis of Metastatic Cancer Patients With Progressive Disease Under Anti-PD-1 Immune Checkpoint Inhibition. Front. Pharmacol..

[21] Romano E., Honeychurch J., Illidge T.M. (2021). Radiotherapy-Immunotherapy Combination: How Will We Bridge the Gap Between Pre-Clinical Promise and Effective Clinical Delivery?. Cancers.

[22] Reynders K., Illidge T., Siva S., Chang J.Y., De Ruysscher D. (2015). The abscopal effect of local radiotherapy: Using immunotherapy to make a rare event clinically relevant. Cancer Treat. Rev..

[23] Arina A., Gutiontov S.I., Weichselbaum R.R. (2020). Radiotherapy and Immunotherapy for Cancer: From “Systemic” to “Multisite”. Clin. Cancer Res..

[24] Brooks E.D., Chang J.Y. (2019). Time to abandon single-site irradiation for inducing abscopal effects. Nat. Rev. Clin. Oncol..

[25] Luke J.J., Lemons J.M., Karrison T.G., Pitroda S.P., Melotek J.M., Zha Y., Al-Hallaq H.A., Arina A., Khodarev N.N., Janisch L. (2018). Safety and Clinical Activity of Pembrolizumab and Multisite Stereotactic Body Radiotherapy in Patients with Advanced Solid Tumors. J. Clin. Oncol..

[26] Frelaut M., Le Tourneau C., Borcoman E. (2019). Hyperprogression under Immunotherapy. Int. J. Mol. Sci..

[27] Champiat S., Dercle L., Ammari S., Massard C., Hollebecque A., Postel-Vinay S., Chaput N., Eggermont A., Marabelle A., Soria J.-C. (2017). Hyperprogressive Disease Is a New Pattern of Progression in Cancer Patients Treated by Anti-PD-1/PD-L1. Clin. Cancer Res..

[28] Iacovelli R., Massari F., Albiges L., Loriot Y., Massard C., Fizazi K., Escudier B. (2015). Evidence and Clinical Relevance of Tumor Flare in Patients Who Discontinue Tyrosine Kinase Inhibitors for Treatment of Metastatic Renal Cell Carcinoma. Eur. Urol..

[29] Ferrara R., Mezquita L., Texier M., Lahmar J., Audigier-Valette C., Tessonnier L., Mazieres J., Zalcman G., Brosseau S., Le Moulec S. (2018). Hyperprogressive Disease in Patients With Advanced Non-Small Cell Lung Cancer Treated With PD-1/PD-L1 Inhibitors or With Single-Agent Chemotherapy. JAMA Oncol..

[30] Wong A.S., Thian Y.L., Kapur J., Leong C.N., Kee P., Lee C.T., Lee M.B. (2018). Pushing the limits of immune-related response: A case of “extreme pseudoprogression”. Cancer Immunol. Immunother..

[31] Baba T., Sakai F., Kato T., Kusumoto M., Kenmotsu H., Sugiura H., Tominaga J., Oikado K., Sata M., Endo M. (2019). Radiologic features of pneumonitis associated with nivolumab in non-small-cell lung cancer and malignant melanoma. Future Oncol..

[32] Wahl R.L., Jacene H., Kasamon Y., Lodge M.A. (2009). From RECIST to PERCIST: Evolving Considerations for PET response criteria in solid tumors. J. Nucl. Med..

[33] Ito K., Teng R., Schöder H., Humm J.L., Ni A., Michaud L., Nakajima R., Yamashita R., Wolchok J.D., Weber W.A. (2019). 18F-FDG PET/CT for Monitoring of Ipilimumab Therapy in Patients with Metastatic Melanoma. J. Nucl. Med..

[34] Goldfarb L., Duchemann B., Chouahnia K., Zelek L., Soussan M. (2019). Monitoring anti-PD-1-based immunotherapy in non-small cell lung cancer with FDG PET: Introduction of iPERCIST. EJNMMI Res..

[35] Cheson B.D., Ansel L., Schwartz L., Gordon L.I., Advani R., Jacene H.A., Hoos A., Barrington S.F., Armand P. (2016). Refinement of the Lugano Classification lymphoma response criteria in the era of immunomodulatory therapy. Blood.

[36] George S., Motzer R.J., Hammers H.J., Redman B.G., Kuzel T.M., Tykodi S.S., Plimack E.R., Jiang J., Waxman I.M., Rini B.I. (2016). Safety and efficacy of nivolumab in patients with metastatic renal cell carcinoma treated beyond progression: A subgroup analysis of a randomized clinical trial. JAMA Oncol..

[37] Escudier B., Motzer R.J., Sharma P., Wagstaff J., Plimack E.R., Hammers H.J., Donskov F., Gurney H., Sosman J.A., Zalewski P.G. (2017). Treatment beyond progression in patients with advanced renal cell carcinoma treated with Nivolumab in CheckMate 025. Eur. Urol..

[38] Mulkey F., Theoret M.R., Keegan P., Pazdur R., Sridhara R. (2020). Comparison of iRECIST versus RECIST V.1.1 in patients treated with an anti-PD-1 or PD-L1 antibody: Pooled FDA analysis. J. Immunother. Cancer.

[39] Seymour L., Bogaerts J., Perrone A., Ford R., Schwartz L.H., Mandrekar S., Lin N.U., Litière S., Dancey J., Chen A. (2017). iRECIST: Guidelines for response criteria for use in trials testing immunotherapeutics. Lancet Oncol..

[40] Bernard-Tessier A., Baldini C., Martin P., Champiat S., Hollebecque A., Postel-Vinay S., Varga A., Bahleda R., Gazzah A., Michot J.M. (2018). Outcomes of long-term responders to anti-programmed death 1 and anti-programmed death ligand 1 when being rechallenged with the same anti-programmed death 1 and anti-programmed death ligand 1 at progression. Eur. J. Cancer.

[41] George G., Schmidt L., Tolat P., Riese M., Kilari D. (2020). Salvage ipilimumab associated with a significant response in sarcomatoid renal cell carcinoma. J. Immunother. Cancer.

[42] Postow M.A., Callahan M.K., Barker C.A., Yamada Y., Yuan J., Kitano S., Mu Z., Rasalan T., Adamow M., Ritter E. (2012). Immunologic correlates of the abscopal effect in a patient with melanoma. N. Engl. J. Med..

