# Supplementary material for: Atypical Response Patterns in Renal Cell Carcinoma Treated with Immune Checkpoint Inhibitors—Navigating the Radiologic Potpourri
Source: Cancers (Basel). 2021 Apr 2;13(7):1689. doi: 10.3390/cancers13071689 (PMC8038243; doi:10.3390/cancers13071689)
Supplement: Supplementary file 1 [file cancers-13-01689-s001.pdf]

Supplementary Materials:

The detailed survival analyses are given here.

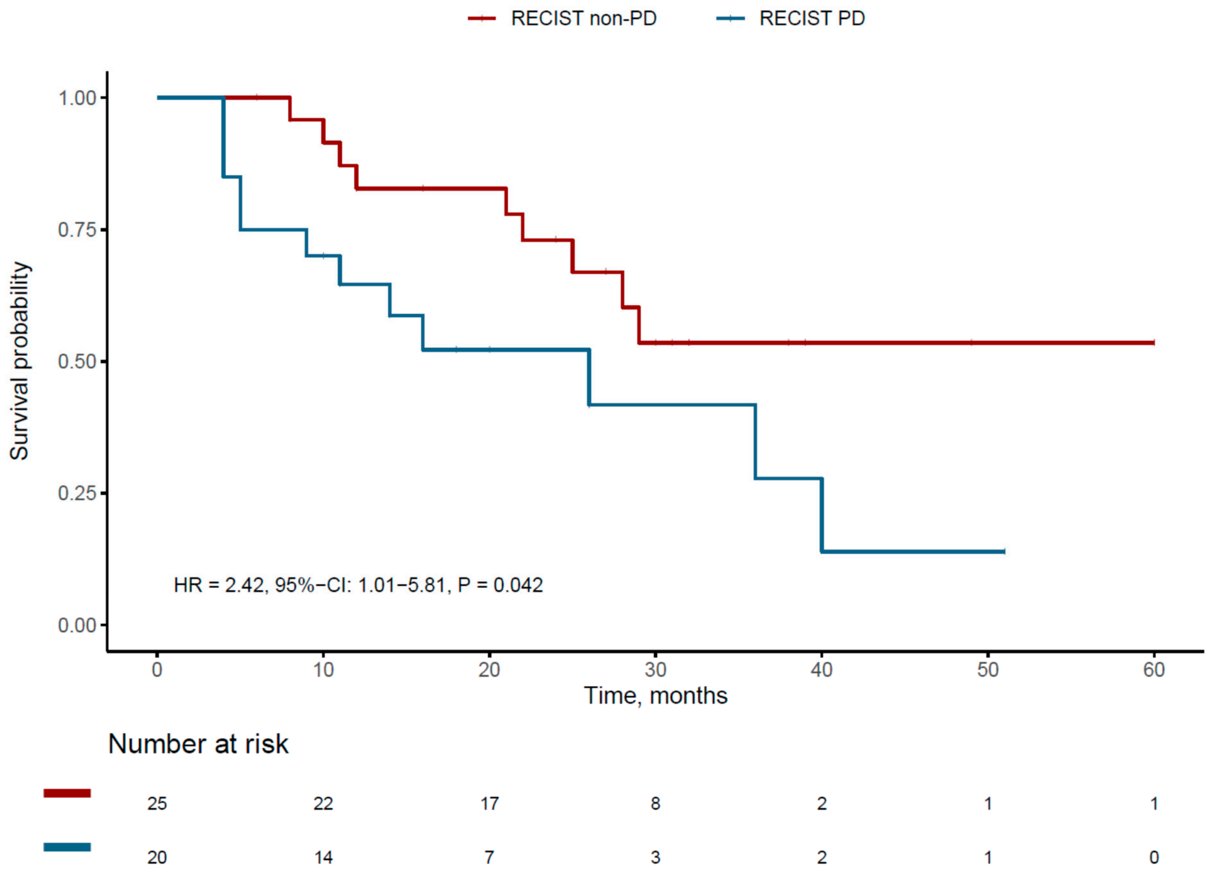

Figure S1. Overall survival by RECIST non-progressive disease versus progressive disease (PD).

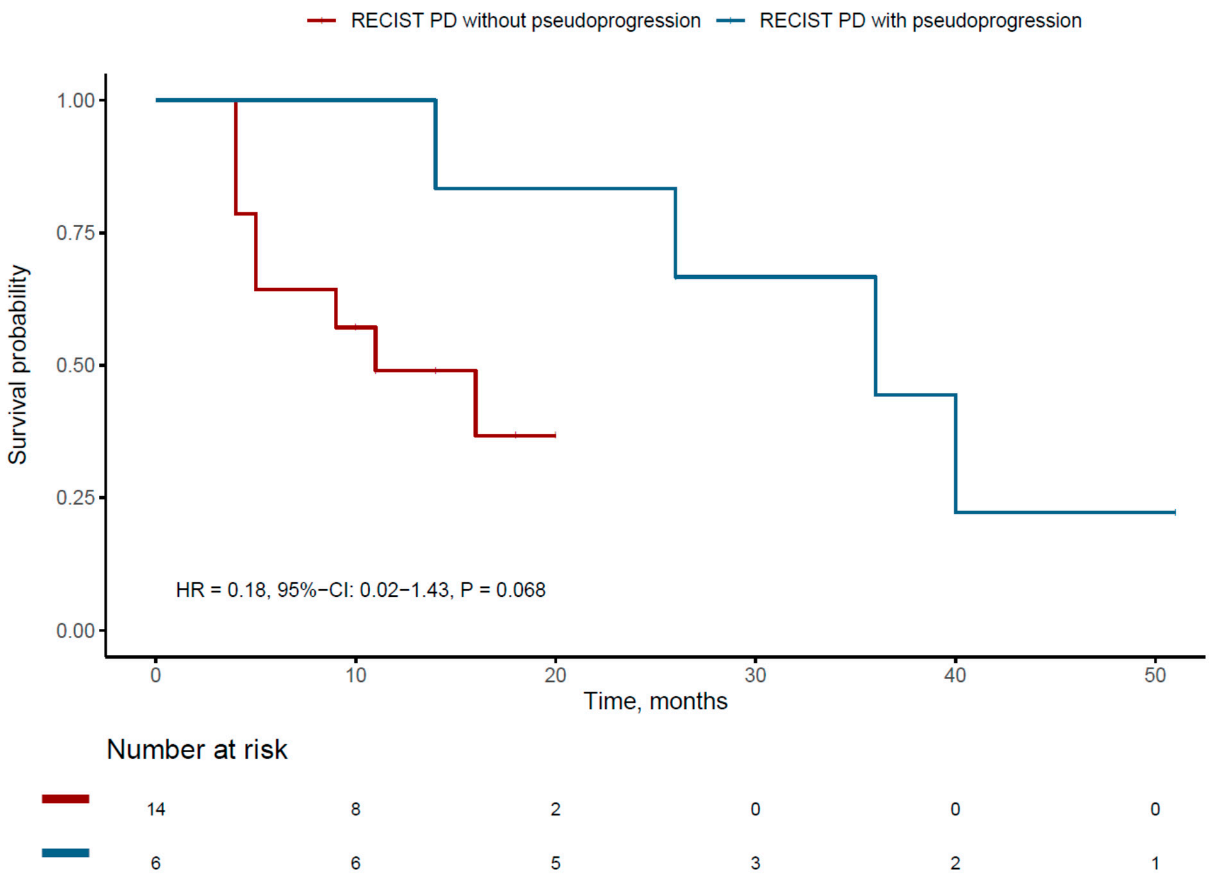

Figure S2. Overall survival in RECIST PD patients by presence or absence of pseudoprogression.

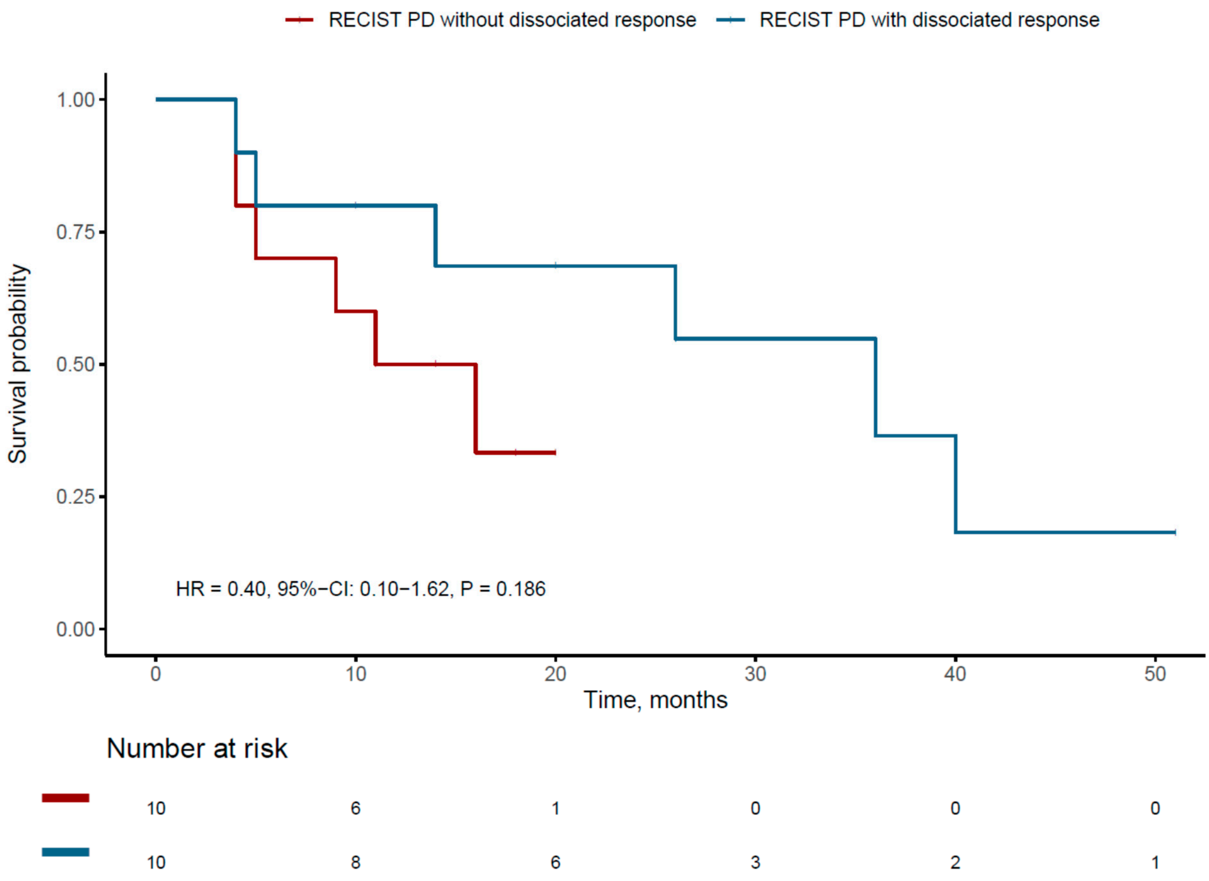

Figure S3. Overall survival in RECIST PD patients by presence or absence of dissociated response.

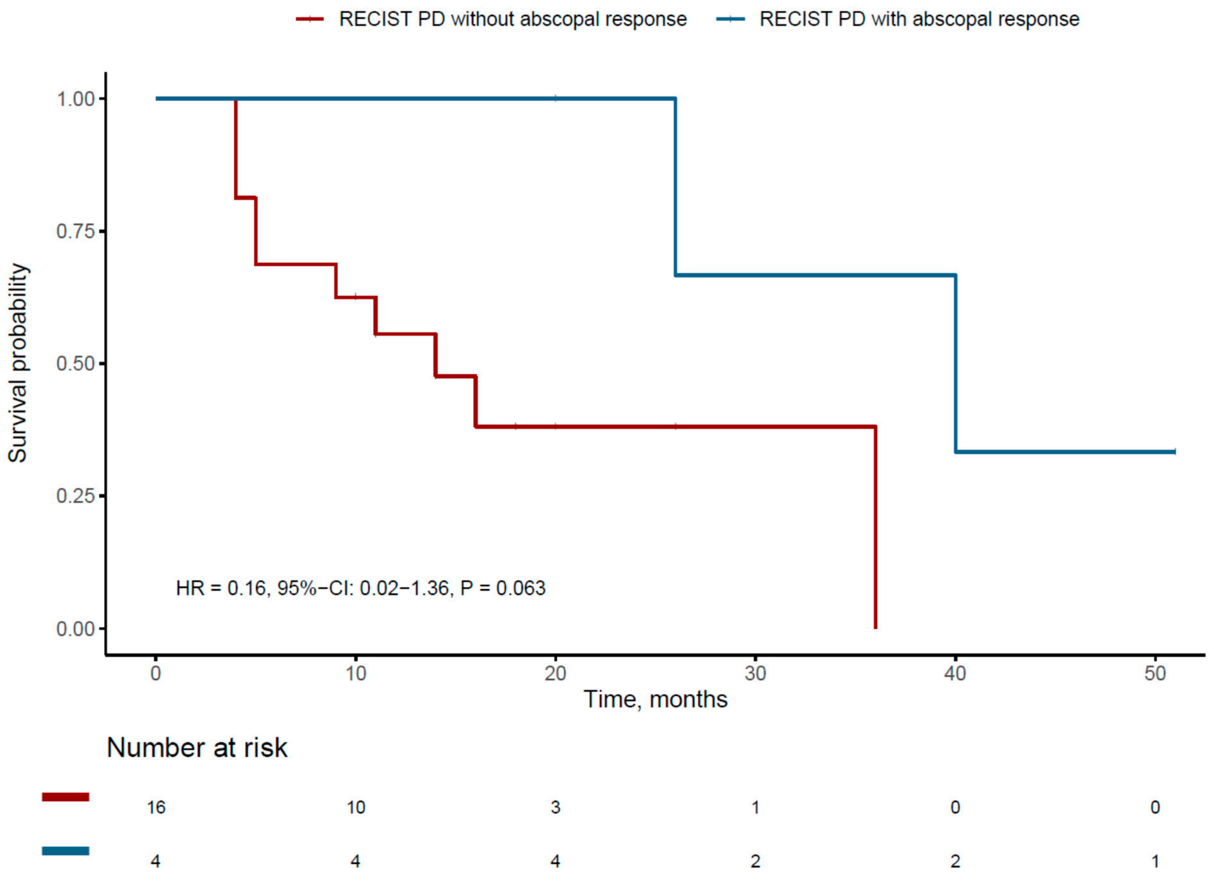

Figure S4. Overall survival in RECIST PD patients by presence or absence of abscopal response.

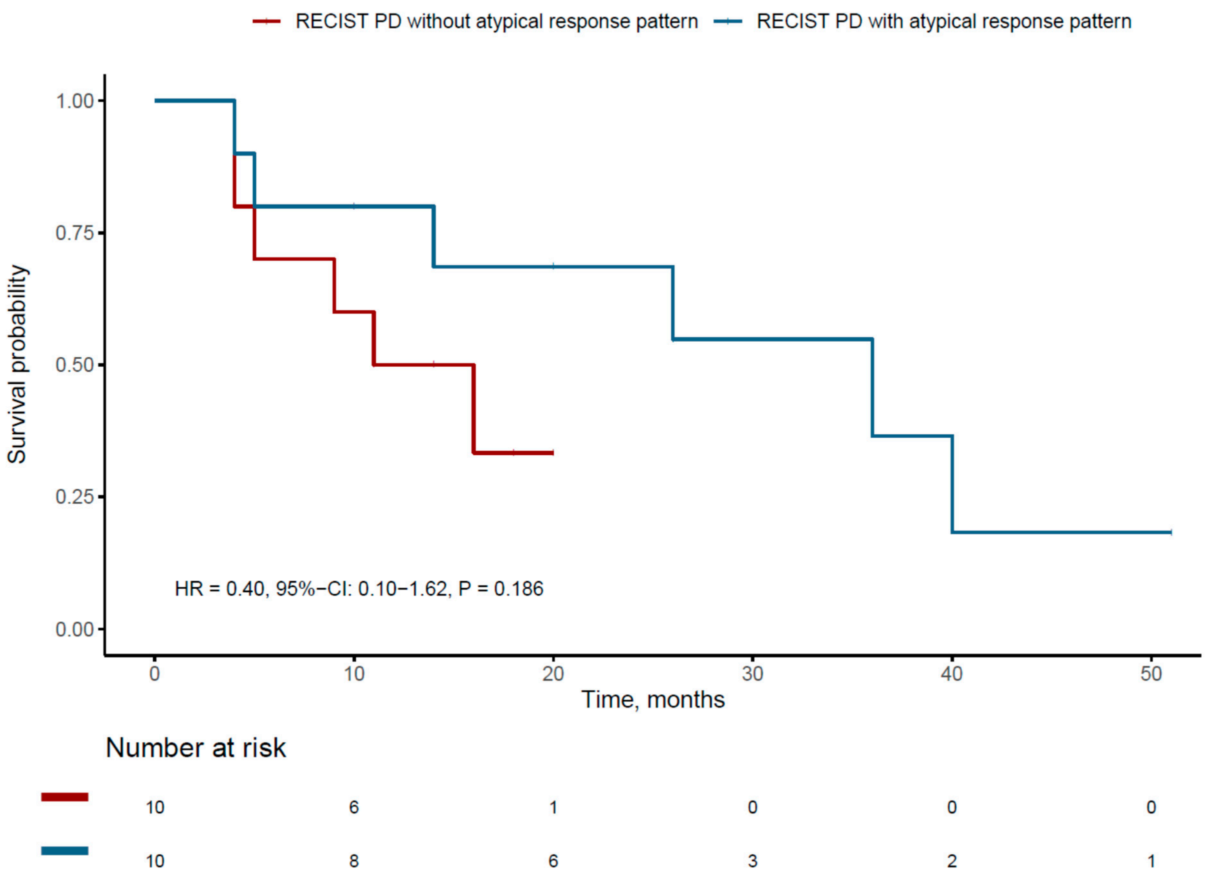

Figure S5. Overall survival in RECIST PD patients by presence or absence of any atypical response pattern.

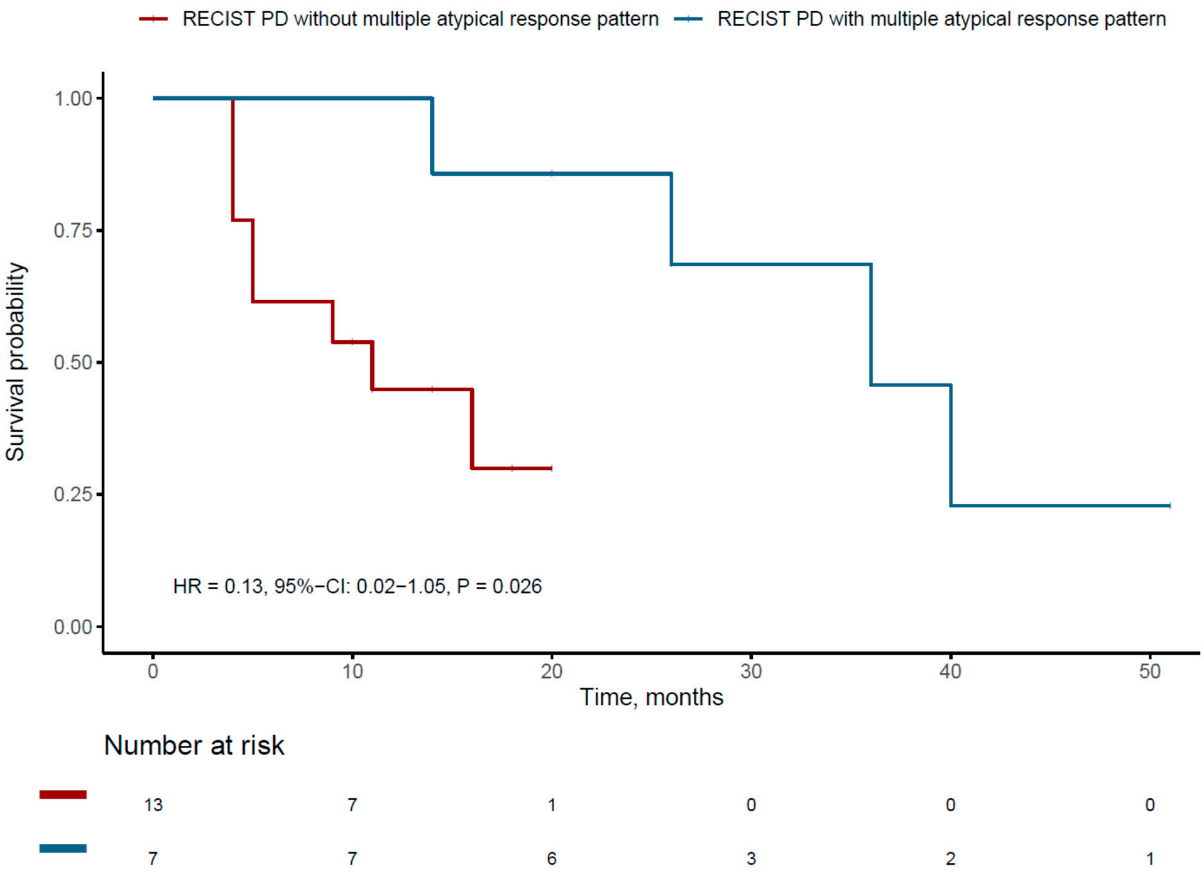

Figure S6. Overall survival in RECIST PD patients by presence or absence of multiple atypical response patterns.
